# International Dental Congress, Paris, 1900

**Published:** 1899-01

**Authors:** 


### INTERNATIONAL DENTAL CONGRESS, PARIS, 1900.

The preliminary report in regard to this important convention has been received. The Congress will take place in Paris during the Exposition.

To prepare for this a convention was held in Paris on the 26th of June, consisting of representatives from the Dental College of Paris, Society of Dentistry of Paris, and from the General Association of France.

A provisional committee was appointed to make arrangements for a permanent organization. Those interested in the Congress and desiring further information should address Dr. E. Pauvez, 17 Rue de Saint-Petersburg, Paris, France.

